# The complete mitochondrial genomes of the two species of *Astyanax* (Characiformes: Acestrorhamphidae) that occur in cenotes of the Yucatán Peninsula karst aquifer: comparative analyses and their taxonomic implications

**DOI:** 10.1007/s11033-025-10788-6

**Published:** 2025-07-10

**Authors:** Jairo Arroyave, Adán Fernando Mar-Silva, Maribel Badillo-Alemán, Maried Ochoa-Zavala

**Affiliations:** 1 Instituto de Biología, Universidad Nacional Autónoma de México, Colección Nacional de Peces, Pabellón Nacional de la Biodiversidad, Cto. Centro Cultural, C.U, Coyoacán, Ciudad de México, 04510 México; 2 Department of Ichthyology, American Museum of Natural History, Central Park West at 79th Street, New York, NY 10024 USA; 3 Facultad de Ciencias, Unidad Multidisciplinaria de Docencia e Investigación, Universidad Nacional Autónoma de México, Puerto de Abrigo s/n, Sisal, México; 4 Escuela Nacional de Estudios Superiores, Unidad Morelia, Universidad Nacional Autónoma de México, Antigua Carretera a Pátzcuaro No. 8701. Col. Ex Hacienda de San José de la Huerta. Morelia, Michoacán Sisal, C.P. 58190 México

**Keywords:** Yucatán tetra, Bacalar tetra, Cenote fishes, Middle American fishes

## Abstract

**Background:**

Despite their high diversity and widespread distribution throughout Neotropical freshwaters, published genomic resources for fishes of the genus *Astyanax* are rather limited. This situation is exemplified by *Astyanax altior* and *Astyanax bacalarensis*, the two species in the genus found in cenotes of the Yucatán Peninsula karst aquifer, a unique yet vulnerable hydrologic system in southeastern Mexico. Distinguishing between these two species based on external morphology, however, is not straightforward, making their purportedly allopatric distributions the most conclusive way to tell them apart. Therefore, testing the current taxonomy is warranted.

**Methods and results:**

To address the perceived deficiency in available genomic data and to test the hypothesis that *A. altior* and *A. bacalarensis* are different species, we generated novel complete mitochondrial genome sequences for both species and analyzed them in a comparative framework. We used a four-step next-generation sequencing protocol consisting of genomic DNA extraction, library preparation, sequencing, and bioinformatic analysis to sequence, assemble and annotate the resulting mitogenomes. Descriptive analyses were used to characterize the mitogenomes, while comparative analyses were used to shed light on the evolutionary relationships and species limits. The complete mitochondrial genomes of *A. altior* and *A. bacalarensis* are almost identical in length, composition, and general gene arrangement, and follow the overall genomic organization of most teleosts. The degree of genetic differentiation between *A. altior* and *A. bacalarensis* was found to be minimal and the hypothesis that they are different species was rejected by our species delimitation analyses.

**Conclusions:**

Besides reporting novel mitogenomic data for a highly diverse group of fishes with limited representation in genomic repositories, our results strongly support previous suspicions that *A. altior* and *A. bacalarensis* correspond to the same species-level lineage and would therefore represent synonyms. Despite our findings, we defer synonymizing them until a proper revisionary study can corroborate this evolutionary and taxonomic hypothesis.

**Supplementary Information:**

The online version contains supplementary material available at 10.1007/s11033-025-10788-6.

## Introduction

*Astyanax* Baird & Girard, 1854 is an extremely speciose and widely distributed Neotropical freshwater fish genus, reported to include, in its broadest sense (*Astyanax sensu lato*), more than 170 species spread from Texas to the *cis*-Andean section of northern Patagonia [1]. Its high diversity and widespread distribution, coupled with a relatively conserved morphology across species, have certainly contributed to its challenging and convoluted systematics and classification, to the extent that, despite its recognition and extensive use, as traditionally understood, it does not constitute a monophyletic taxon [1–3]. A recent phylogenetic study of the genus by Terán et al. [1], however, offers a preliminary redefinition to the limits and composition of a monophyletic *Astyanax*, consisting of a considerably less diverse clade that includes the type species of the genus (*A. argenteus*) and the Central and North American species. Although historically classified in the family Characidae, *Astyanax*—as defined by Terán et al. [1]—was recently reclassified as a member of Acestrorhamphidae, a family-level clade discovered by Melo et al. [4] in a higher-level phylogenomic study of characid fishes.

Within this reduced *Astyanax sensu* Terán et al. [1] (hereafter *Astyanax sensu stricto* [*s.s.*]), there are two species, *Astyanax altior* Hubbs 1936 and *Astyanax bacalarensis* Schmitter-Soto 2017, that occur in a unique hydrological system: the karst aquifer of the Yucatán Peninsula (YP) in southeastern Mexico [5, 6]. This region is characterized by the presence of thousands of karst sinkholes, locally known as cenotes, which are essentially openings in the limestone from which water from the underlying aquifer (the Gran Acuífero Maya) reaches the surface, thus becoming readily available for terrestrial organisms from the surrounding forest and for the establishment of aquatic communities [7–9]. These cenotes and their associated submerged karstic caves are home to a variety of aquatic fauna, including many endemics [9].

Both *A. altior* and *A. bacalarensis* were proposed in a revisionary study of Central and North American tetras of the genus *Astyanax*, with the former resurrected and redescribed and the latter newly described [10]. The Yucatán tetra, *A. altior*, is a species geographically restricted (endemic) to northwestern Yucatán in Mexico, where it primarily inhabits karstic springs of varying configuration and associated landscape and vegetation, such as cenotes, aguadas (old cenotes with stagnant water as a result of sediments and debris interrupting water flow), and petenes (tree-covered islands in coastal wetlands fed by groundwater seeping up from cenotes) [6]. Conversely, the Bacalar tetra, *A. bacalarensis*, has a broader distribution and range of habitats, occurring along the Caribbean versant of the YP with records from the Mexican state of Quintana Roo (e.g., cenotes of the Riviera Maya, Bacalar Lake), Belize (e.g., Sittie River), and a small portion of northern Guatemala (e.g., Mopán River) [5, 10].

Despite the species richness of the group, published genomic resources for *Astyanax* are relatively rather limited, among which there are a few whole genomes and transcriptomes of surface and cave-dwelling populations of *A. mexicanus* [11–13] and a few complete mitochondrial genomes [14–17], most of which, however, from species of *Astyanax sensu lato*. Contributing to the sparse comparative genomic data available for *Astyanax* is therefore important and even necessary for future systematics research as well as other lines of evolutionary inquiry. Phenotypically, *A. bacalarensis* and *A. altior* are very similar, and distinguishing between the two using external morphology is not straightforward (due to overlapping and rather variable diagnostic characters), making their purportedly allopatric distributions the most conclusive way to tell them apart. The main external morphological characters used to differentiate between these two species are body depth, head length, position of anal fin with respect to last dorsal-fin ray, and scales between lateral line and dorsal-fin origin ray [10].

This issue of problematic diagnoses was similarly raised by Terán et al. [1], but in a broader sense, when referring to the identification of Central and North American species of *Astyanax* following the revision of Schmitter-Soto [10]. Generation and phylogenetic analysis of molecular comparative data from *A. altior* and *A. bacalarensis*, such as complete mitochondrial genomes, can therefore test the validity of these species with molecular data in a phylogenetic framework. Thus, to shed light on the systematics and evolutionary genomics of *Astyanax* from Middle America, in this study we present the first complete mitochondrial genomes—fully assembled and annotated—of the YP-endemic tetras *A. altior* and *A. bacalarensis*, accompanied by detailed descriptive (genome size and organization, protein-coding genes [PCGs], non-coding regions, and RNAs features) and comparative (patterns of selection on PCGs, tree estimation, and species delimitation) analyses.

## Methods

### Specimen sampling

Specimen vouchers from *A. altior* (CNPE-IBUNAM 24452) and *A. bacalarensis* (CNPE-IBUNAM 23833) used to generate the complete mitochondrial genomes reported herein were collected from Petén Pila (a karstic spring near Sisal, Yucatán, Mexico; 21°09’31.9"N 90°02’22.5"W) and Lake Bacalar (Quintana Roo, Mexico; 18°40’31"N 88°23’17.89"W), respectively. Fishes were collected, handled, and euthanized (with MS-222) prior to preservation in accordance with recommended guidelines for the use of fishes in research [18], and under collecting permits SGPA/DGVS/05375/19 and SGPA/DGVS/08073/21 issued by the Secretaría de Medio Ambiente y Recursos Naturales (SEMARNAT) to JA. Tissue samples (fin clips) were taken prior to specimen fixation, preserved in 95% ethanol, and eventually stored frozen at -20 °C. After tissuing, specimens were fixed in a 10% formalin solution and later transferred to 70% ethanol for long-term storage in the national fish collection (Colección Nacional de Peces [CNPE], Instituto de Biología [IB], Universidad Nacional Autónoma de México [UNAM]). Samples were identified to species using the taxonomic key proposed by Schmitter-Soto [10], with species identifications corroborated based on geographic distribution (*A. altior* and *A. bacalarensis* being allopatric). The sample from *A. bacalarensis* is a topotype, whereas that from *A. altior* is from a locality just ~ 40 km in linear distance from its type locality.

### NGS library Preparation and sequencing

High-molecular genomic DNA was extracted from fresh tissue samples using the phenol-chloroform protocol [19]. Genomic DNA was fragmented via sonication using a Diagenode Bioruptor^®^ Pico sonication device for 6 cycles of alternating 30 s ultrasonic bursts and 30 s pauses in a 4 °C water bath. Library preparation was carried out from a total of 200 ng of fragmented DNA per sample (quantified using a Qubit fluorometer [Invitrogen]) using the KAPA HyperPrep Kit (Kapa Biosystems Inc., Wilmington, MA), which employs the KAPA HiFi DNA Polymerase, a B-family DNA polymerase engineered for increased processivity, low bias, and high-fidelity library amplification. Fragmented DNA was end-repaired and end-polished via A-tailing, and subsequently ligated to custom TruSeq dual-indexed adapters [20] via short PCR. Products were size selected in a ~ 300–500 bp range (standard fragment length for optimal performance when sequencing using the Illumina™ HiSeq X system) using magnetic beads, enriched through PCR (using the abovementioned kit), purified, and normalized. Mitogenome libraries were sequenced in an Illumina™ HiSeq X at an average sequencing depth of 50X, resulting in paired-end sequences of 150 bp in length (standard read length of the HiSeq X sequencing platform). Illumina™ sequencing was conducted at the Georgia Genomics and Bioinformatics Core of the University of Georgia (Athens, GA).

### Mitogenome assembly and annotation

Raw data quality was first evaluated in FastQC [21], after which paired reads were trimmed using Geneious Prime v2024.0.3 (https://www.geneious.com). Mitogenome assembly was carried out using the module “map to reference” in Geneious, using three complete mitochondrial genomes of *Astyanax* available in GenBank (*A. aeneus* accession BK013055 [16], *A. mexicanus* accessions BK013062 [16] and AP011982 [15]) as reference genomes for mapping reads during assembly. These mitogenomes were used one at a time during assembly, thus generating three consensus mitogenomes for each of the two novel mitogenomes reported here.

Consensus mitogenomes of each species (*A. altior* and *A. bacalarensis*) were then aligned in MEGA X [22] to obtain the final mitogenome of each species. Identification and annotation of the 37 mtDNA genes (two rRNAs, 22 tRNAs and 13 polypeptides) and non-coding control region (including their strand position) of each newly generated mitochondrial genome was accomplished using MitoFish and MitoAnnotator [23].

### Descriptive analyses

Nucleotide and amino acid composition, codon usage profiles of protein-coding genes (PCGs), Relative Synonymous Codon Usage (RSCU), and characterization the non-coding mtDNA control region (CR) were computed with MEGA X [22]. Nucleotide composition skewness was calculated with the formulas AT skew = (A − T)/(A + T) and GC skew = (G − C)/(G + C) [24]. Prediction of tRNAs secondary structure was accomplished with tRNAScan-SE 2.0 [25] through its webserver (https://lowelab.ucsc.edu/tRNAscan-SE/) using Infernal without HMM filter search mode and “vertebrate mitochondrial” as sequence source [26]. Analysis and prediction of CR secondary structure was carried out with the software ClustalW [27] as implemented in MEGA X [22] by comparison (via multiple sequence alignment) with reports of secondary CR structure from the teleost *Siniperca chuatsi* (GenBank accession number EU659698) [28].

### Comparative analyses

Patterns of genetic differentiation and phylogenetic relationships in *A. altior* and *A. bacalarensis* were investigated based on mitogenome-wide variation in a comparative framework. For the phylogenetic component, complete mitochondrial genomes from *Astyanax s.s.* available in GenBank (i.e., *A. aeneus* [accession number BK013055], *A. mexicanus* [accession numbers BK013062 and AP011982], *A. altiparanae* [accession numbers MT428072 and MN583176], and *A. lacustris* [accession number MT428067]) were sampled as part of the ingroup, whereas a selection of genera closely related to *Astyanax s.s.* with available complete mitogenomes (*Psalidodon* [*P. fasciatus*: accession number OP218849, *P. paranae*: accession number NC_031380], *Paracheirodon* [*P. axelrodi*: accession numbers B898197 and MH998225], and *Deuterodon* [*D. giton*: accession number MF805815]), were sampled as part of the outgroup, with *D. giton* as root.

Complete mitogenomes were aligned using MUSCLE [29] as implemented in Mega X [22]. A subset of mitogenomes from the Middle American species of *Astyanax s.s.* (*A. aeneus*, *A. mexicanus*, *A. altior*, and *A. bacalarensis*) was used to assess phenetic patterns of genetic variation of the newly generated mitogenomes with respect to those of close relatives. Mitogenome-level genetic divergences among samples from this subset were estimated and visualized via *p*-distances using Mega X [22] as well as mean Kimura-2-parameter (K2P) distances for 60 bp windows using the approach and customized R script of Santos et al. [30], using a multiple sequence alignment of ingroup mitogenomes as input data and the sample from *Astyanax altior* (GenBank accession number PV765696) as the base for comparisons.

Phylogenetic relationships were investigated using Bayesian approaches based on a concatenated alignment of the 13 mitochondrial protein-coding genes (PCGs). For this data matrix, GTR + G was selected as the best-fit model of nucleotide substitution according to the corrected Akaike Information Criterion (AICc) as implemented in jModelTest v2.1.10 [31] (-lnL = 44414.86613, AICc = 88908.005896). Bayesian inference of phylogeny was first carried out in MrBayes v3.2.6 [32] using the Markov Chain Monte Carlo algorithm (MCMC) run for 10^6^ generations with a sampling period of 500 generations under default priors and default proposal mechanisms, with a total of two independent runs of four chains each. Convergence of the MCMC algorithm to a stationary distribution—and thus the number of generations to be discarded as burn-in—was determined by examination of trace plots of posterior probability vs. number of generations using Tracer [33]. Accordingly, 10% of MCMC samples were discarded as burn-in, and substitution model parameters were calculated from the remaining 90%. Likewise, branch lengths and posterior probabilities of nodes were calculated from the set of post burn-in trees using TreeAnnotator [34] and summarized as a 50% majority rule consensus tree.

To account for confounding issues leading to gene-tree/species-tree discordance [35, 36] and to assess species limits, an additional Bayesian analysis based on the multispecies coalescent was conducted on the same data matrix using the software bpp v4.8.2 [37]. Species delimitation using bpp was implemented on a user-specified guide tree (guided; A10 analysis) [38] and as a joint species delimitation and species tree inference procedure (unguided; A11 analysis) [39, 40]. The phylogeny inferred with MrBayes was used as the guide tree for the A10 analysis. Both A10 and A11 analyses were run using the reverse-jump Markov Chain Monte Carlo (rjMCMC) algorithms 0 and 1 [38], with automatically optimized step lengths for rjMCMC proposals, set for 500,000 generations sampled every two generations, and with 20,000 samples discarded as burn-in. Priors for ancestral population size (θ) and divergence times (τ) for the root of the species tree were both modeled by a gamma distribution (a = 2 and b = 2000), with the remaining species divergence times generated from a uniform Dirichlet distribution [38]. Each analysis was run twice with different starting seeds to verify consistency between runs. Each algorithm in each run produced acceptance proportions neither too small (< 0.20) not too large (> 0.7), thus assuring efficiency of the MCMC. The matrix analyzed contained a total of 13 terminals (including the root outgroup, *Deuterodon giton*) from 10 species (coded as populations A–J in the Imap bpp input file) to be tested in the species delimitation analyses. Although *Astyanax altiparanae* is considered a junior synonym of *Astyanax lacustris* by some authors [41, 42], we made the distinction between these two species in our data matrix following the species identities reported in GenBank. In this way, although tangential to our main research question, we were able to test this synonymy in the context of our species delimitation analyses, leveraging the fact that bpp never tries to split one population into multiple species, although it does attempt to merge different populations into one species [43]. Overall, the A10 and A11 analyses tested 85 different models of species delimitation.

## Results

### Mitogenome size and organization

High throughput sequencing of mitogenomic libraries resulted in 14,274,348 and 14,912,484 reads for the samples of *Astyanax altior* and *Astyanax bacalarensis*, respectively. These raw data are available as an NCBI (https://www.ncbi.nlm.nih.gov/bioproject/) BioProject (PRJNA1276058). FastQC quality reports in HTML format (clean data), as well as Geneious assemblies’ reports, are available in the free, open-source, general-purpose data repository Zenodo (https://www.zenodo.org) (10.5281/zenodo.15647728).

**Fig. 1:**
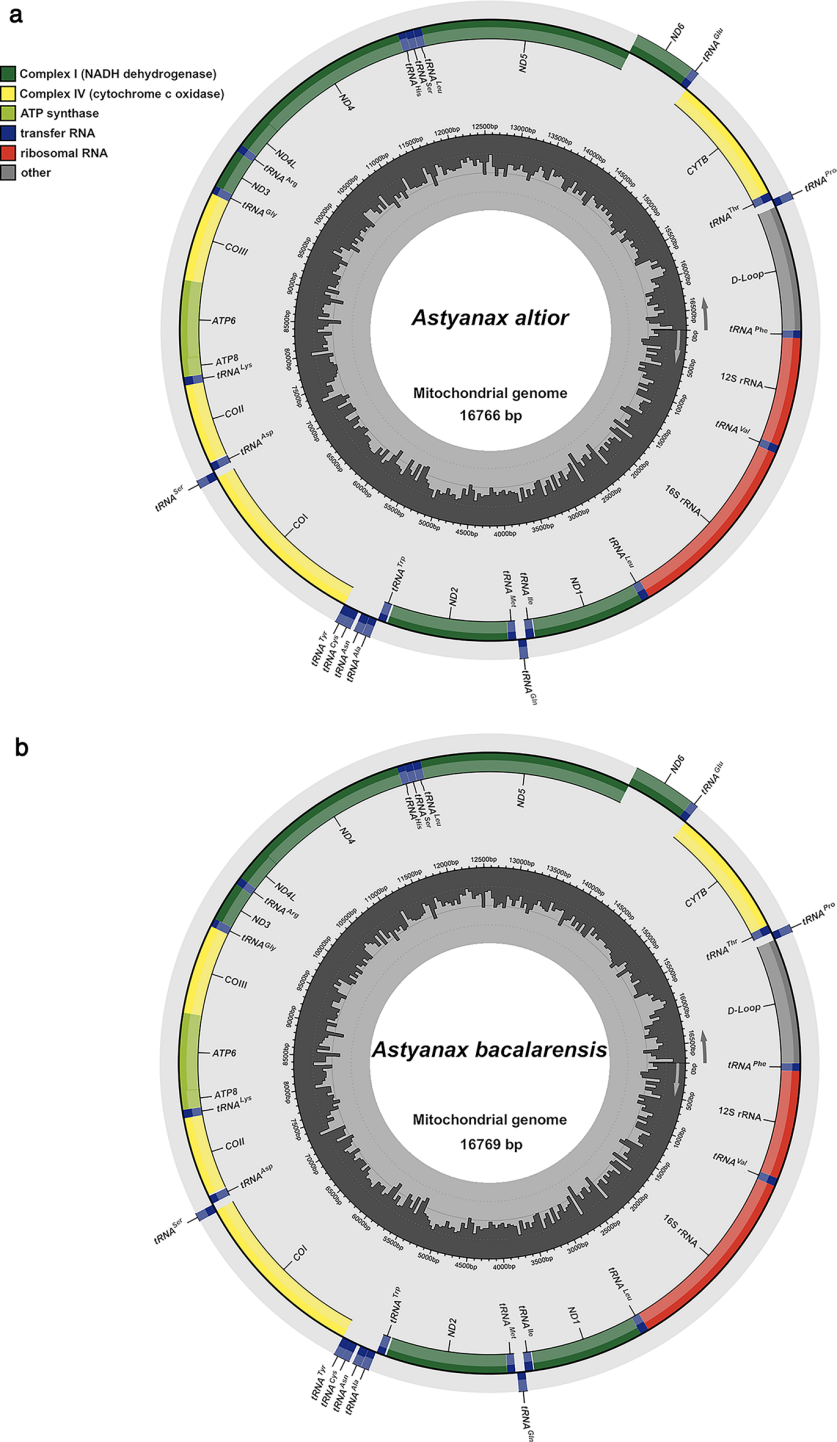
Annotated map of the mitochondrial circular genome of (a) *Astyanax altior* (CNPE-IBUNAM 24452, GenBank accession number PV765696) and (b) *Astyanax bacalarensis* (CNPE-IBUNAM 23833, GenBank accession number PV765695). The outer ring corresponds to the H- (outermost) and L-strands, and depicts the location of PCGs, the non-coding control region (D-loop), tRNAs, and rRNAs. The inner ring (black sliding window) denotes GC content along the genome

The complete mitochondrial genomes of *Astyanax altior* (GenBank accession number PV765696) and *Astyanax bacalarensis* (GenBank accession number PV765695) are almost identical in total length, being 16,766 bp and 16,769 bp, respectively (Table 1; Fig. 1). The composition and general arrangement of the mitochondrial genes in both species conforms to that reported for other teleost fishes, including other species of the genus *Astyanax*, consisting of a total of 37 genes categorized as follows: 13 PCGs, 2 rRNAs, 22 tRNAs and one non-coding Control Region (CR) that includes a displacement loop (D-Loop) region (Fig. 1). Twenty-eight genes (12 PCGs, 2 rRNAs, and 14 tRNAs) plus the CR are encoded on the H-strand, while the remaining nine genes are found on the L-strand (*NAD6*, and 8 tRNAs) (Table 1). The mitogenomes of *A. altior* and *A. bacalarensis* have similar numbers of intergenic spacers (IGS): 15 (totaling 114 bp) and 14 (totaling 123 bp), respectively, and ranging between 1 and 30 bp in length. The longest IGS for both species (30 bp) is found between *tRNA*^*Ala*^ and *tRNA*^*Cys*^, which corresponds to the L-strand origin of replication (OL) (Table 1). Both mitogenomes exhibit a total of seven regions with overlapping genes (negative IGS values in Table 1), the longest being 11 bp between *COI* and *tRNA*^*Ser*^. The overall base composition (A = 30%, T = 28.9%, G = 15.4%, and C = 25.7%) and GC-content (41%) turned out to be the same for both mitogenomes. Likewise, both mitogenomes exhibit positive AT and negative GC skewness, with almost identical values (0.0190 vs. 0.0184 and − 0.250 vs. -0.249, respectively).

**Table 1:** Mitochondrial genes and associated features of *Astyanax altior* and *Astyanax bacalarensis*. Values in parentheses correspond to *A. bacalarensis* when different from those in *A. altior*. Intergenic space (IGS) described as intergenic (+) or overlapping nucleotides (–). AA = amino acid

Locus	Code	Start	End	Length (bp)	Strand	Total AA	Anticodon	Start codon	Stop codon	IGS
tRNAPhe	F	1	68	68	H		GAA			0
*12s rRNA*		69	1016	948	H					0
tRNAVal	V	1017	1088	72	H		TAC			0
*16s rRNA*		1089	2758	1670	H					0
tRNALeu1	L	2759	2833	75	H		TAA			0
*NAD1*		2834	3805	972	H	323		ATG	TAA	10
tRNAIle	I	3814	3885	72	H		GAT			-2
tRNAGln	Q	3884	3954	71	L		TTG			15
tRNAMet	M	3970	4039	70	H		CAT			2
*NAD2*		4042	5094 (5097)	1053 (1056)	H	350 (351)		ATG	TAA	16
tRNATrp	W	5111 (5114)	5181 (5184)	71	H		TCA			8
tRNAAla	A	5189 (5192)	5257 (5260)	69	L		TGC			1
tRNAAsn	N	5259 (5262)	5331 (5334)	73	L		GTT			30
OL					H					
tRNACys	C	5362 (5365)	5429 (5432)	68	L		GCA			-1
tRNATyr	Y	5429 (5432)	5499 (5502)	71	L		GTA			1
*COI*		5501 (5504)	7060 (7063)	1560	H	519		GTG	AGG	-11
tRNASer1	S	7048 (7051)	7119 (7122)	72	L		TGA			10 (8)
tRNAAsp	D	7128 (7131)	7199 (7202)	72	H		GTC			14 (24)
*COII*		7214 (7217)	7904 (7907)	691	H	230		ATG	T	14 (0)
tRNALys	K	7905 (7908)	7977 (7980)	73	H		TTT			1
*ATP8*		7979 (7982)	8146 (8149)	168	H	55		ATG	TAA	-9
*ATP6*		8137 (8140)	8818 (8821)	682	H	227		ATG	TA	0
*COIII*		8819 (8822)	9602 (9605)	784	H	261		ATG	T	0
tRNAGly	G	9603 (9606)	9675 (9678)	73	H		TCC			0
*NAD3*		9676 (9679)	10,024 (10027)	349	H	116		ATG	T	0
tRNAArg	R	10,025 (10028)	10,093 (10096)	69	H		TCG			0
*NAD4L*		10,094 (10097)	10,390 (10393)	297	H	98		ATG	TAA	-6
*NAD4*		10,384 (10387)	11764 (11767	1381	H	460		ATG	T	0
tRNAHis	H	11,765 (11768)	11,833 (11836)	69	H		GTG			0
tRNASer2	S	11,834 (11837)	11,901 (11904)	68	H		GCT			1
tRNALeu2	L	11,903 (11906)	11,974 (11977)	72	H		TAG			0
*NAD5*		11,975 (11978)	13,813 (13816)	1839	H	612		ATG	TAA	-4 (-3)
*NAD6*		13,810 (13813)	14,325 (14328)	516	L	171		ATG	TAA	0
tRNAGlu	E	14,326 (14329)	14,393 (14396)	68	L		TTC			6 (5)
*Cyt b*		14,399 (14402)	15,535 (15538)	1137	H	378		ATG	TAA	1
tRNAThr	T	15,537 (15540)	15,608 (15611)	72	H		TGT			-1 (-2)
tRNAPro	P	15,607 (15610)	15,675 (15678)	69	L		TGG			0
*D-loop*		15,676 (15679)	16,766 (16769)	1091	H					0

### Protein-coding genes

**Fig. 2:**
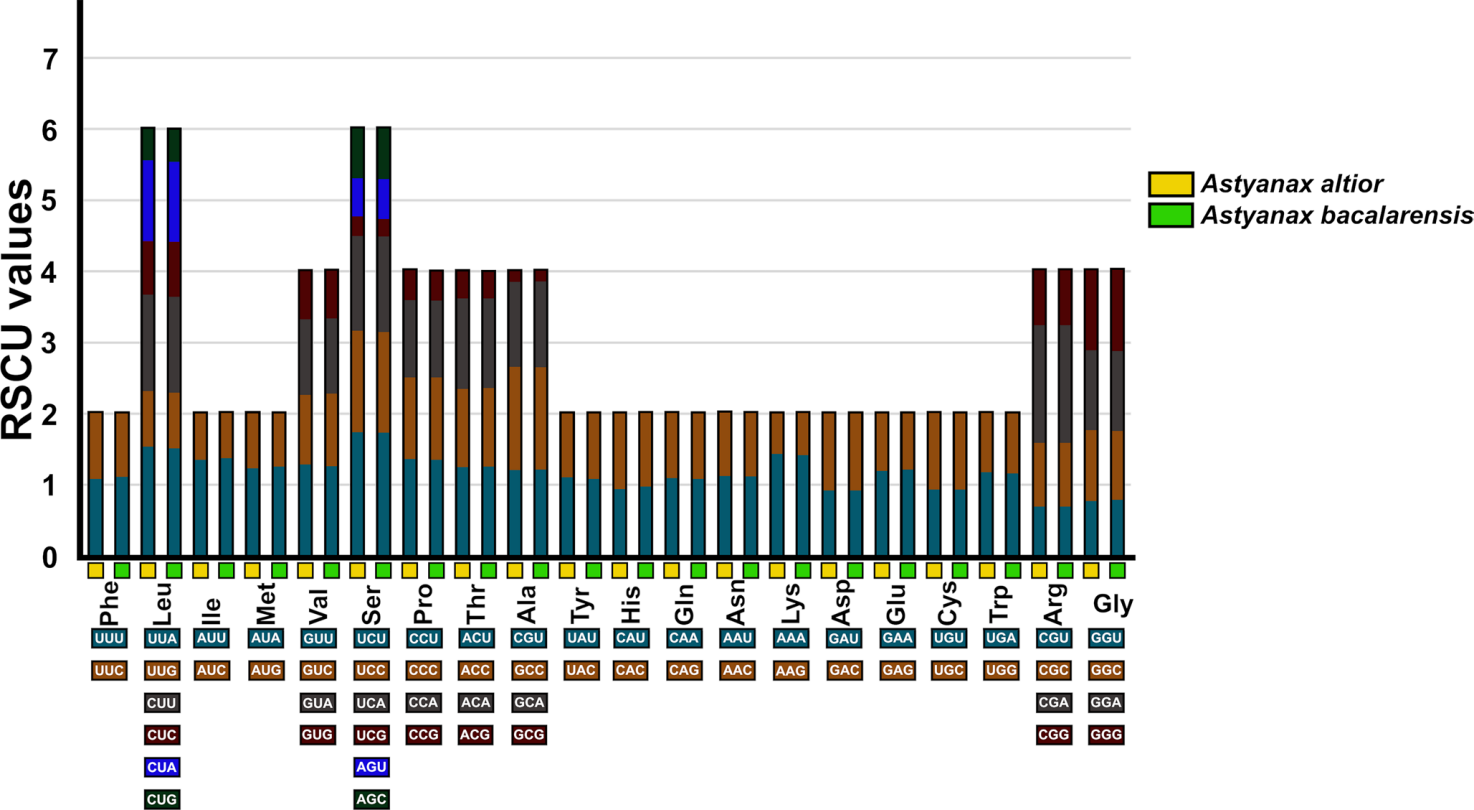
Results from analysis of Relative Synonymous Codon Usage (RSCU) of the mitochondrial genomes of *Astyanax altior* and *Astyanax bacalarensis*. Codon families are plotted on the x-axis. The label for the 2, 4, or 6 codons that compose each family is shown in the boxes below the x-axis, and the colors correspond to those in the stacked columns. RSCU values are shown on the y-axis

In both species, the 13 PCGs altogether encompass 68.1% of the mitogenome, totaling almost the same number of nucleotides (11,429 and 11,432 bp in *A. altior* and *A. bacalarensis*, respectively). Like in most animal mitogenomes, these genes comprise seven regions that code for the subunits of the NADH dehydrogenase (ubiquinone) protein complex (*NAD1-6*, *NADL4*), three that code for the subunits of the enzyme cytochrome c oxidase (*COI-III*), one that codes for the enzyme cytochrome b (*CYTB*), and two that code for the subunits 6 and 8 of the enzyme ATP synthase FO (*ATP6*, *ATP8*). Except for *COI*, PCGs exhibit an ATG (Met) start codon, which is the standard in eukaryotic systems [44]; the start codon in *COI* (GTG), however, is fairly common among vertebrates [45] (Table 1). Both mitogenomes display the same pattern of stop codons, consisting of seven PCGs (*NAD1*, *NAD2*, *ATP8*, *NAD4L*, *NAD5*, *NAD6* and *CYTB*) terminating with TAA, five PCGs (*COII*, *ATP6*, *COIII*, *NAD3*, and *NAD4*) with an incomplete stop codon (either TA or T), and *COI* with the noncanonical stop codon AGG. Consistent with the high similarity in composition and organization of PCGs between mitogenomes, they encode almost the same number of amino acids (3,608 and 3,612 in *A. altior* and *A. bacalarensis*, respectively), with Leu being the most frequent (15.4%) and Cys and Arg (1.7%) the rarest. Results from RSCU analysis of PCGs are virtually the same for both mitogenomes (Fig. 2) and indicate that UCU (encoding Ser) is the most common codon (1.74%) whereas GCG (encoding Ala) is the least frequent (0.14%).

### RNAs and non-coding DNA

The mitogenomes of *A. altior* and *A. bacalarensis* contain the usual 22 tRNAs characteristic of mitogenomes from other teleosts and vertebrates, with fourteen tRNAs encoded in the H-strand and eight in the L-strand (Table 1, Fig. 1). Altogether, tRNAs total 1,557 bp, with individual ones ranging from 68 (*tRNA*^*Phe*^, *tRNA*^*Cys*^, *tRNA*^*Ser2*^, and *tRNA*^*Glu*^) to 75 bp (*tRNA*^*Leu*^) in both mitogenomes (Table 1). Both mitogenomes have the same tRNAs nucleotide composition (A = 28.8%, T = 27.6%, C = 20%, and G = 23.6%) and CG-content (43.6%). Except for *tRNA*^*Ser1*^, in which the D arm is missing in both mitogenomes, all tRNAs fold into the canonical cloverleaf secondary structure that consists of four domains (AA stem, D arm, AC arm, and T arm) and a variable loop (Figs. S1 and S2). In both species, the genes that code for the mitochondrial 12S and 16S rRNA subunits are 948 and 1670 bp long, respectively, and like in most teleost fishes, located on the H-strand and separated by the *tRNA*^*Val*^ (Table 1). Like with tRNAs, both mitogenomes exhibit virtually the same rRNAs nucleotide composition (A = 34.1%, T = 22.7%, C = 23.3%, and G = 19.9%) and CG-content (43.2%). The mtDNA control region of both *A. altior* and *A. bacalarensis* is 1091 bp long and flanked by *tRNA*^*Pro*^ and *tRNA*^*Phe*^ at the 5’ and 3’ ends, respectively. As with the overall genome and both tRNAs and rRNAs, nucleotide composition (A = 35.3%, T = 31.1%, C = 20.6%, and G = 13.0%) and CG-content (43.6%) of the CR are effectively equivalent in both mitogenomes.

**Fig. 3:**
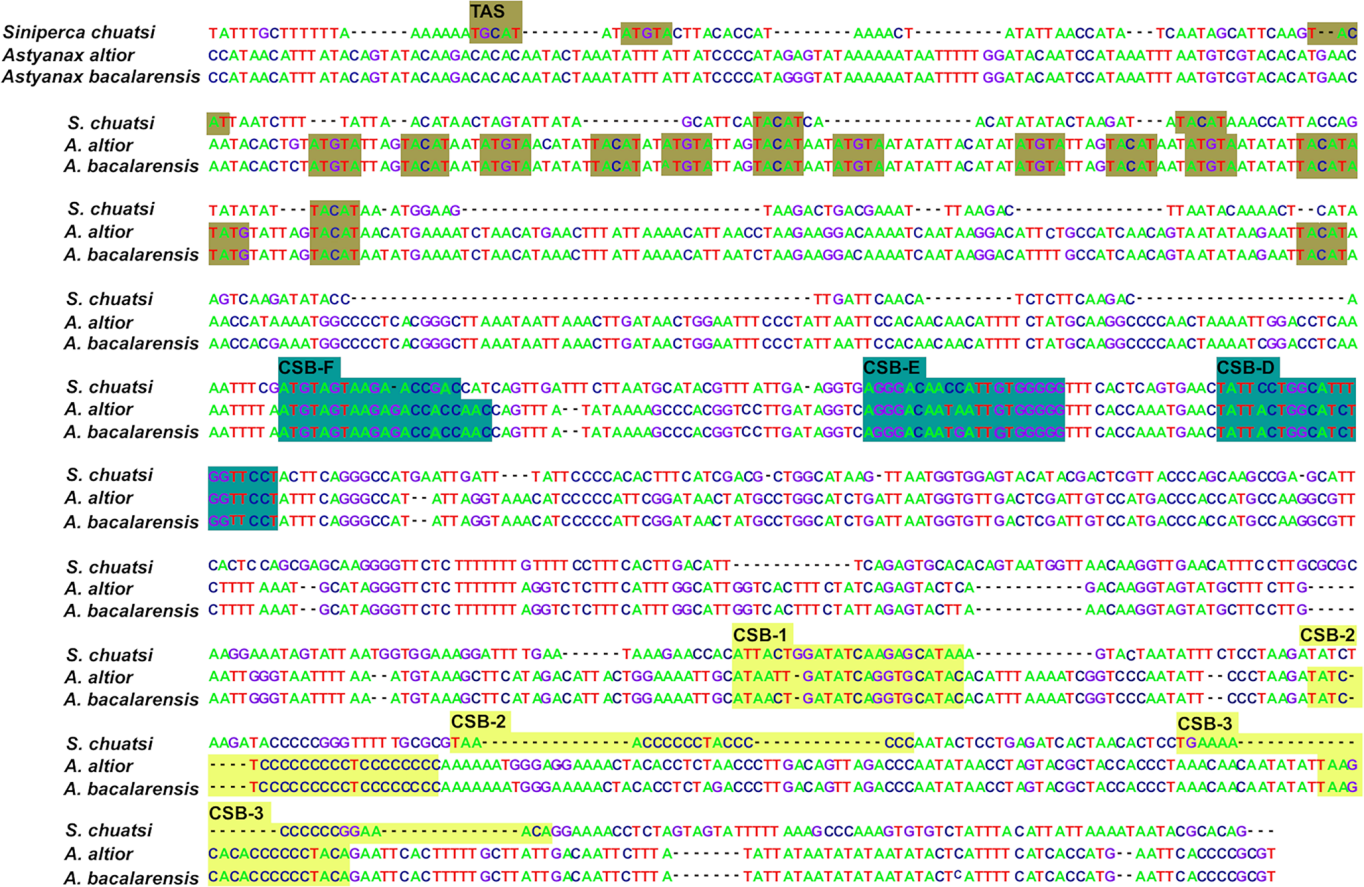
Comparison (multiple sequence alignment) of the mtDNA control region of *Astyanax altior* and *Astyanax bacalarensis* with respect to that of the teleost *Siniperca chuatsi*. The alignment displays the three canonical domains distinguished by Termination Associated Sequences (TAS) of the upstream hypervariable region (in gold), central conserved domain blocks (CSB-F, CSB-E, CSB-D) (in green), and conserved sequence blocks of the downstream hypervariable region (CSB-1, CSB-2 and CSB-3) (in yellow)

Like in other fishes, CR in *Astyanax* is divided into three domains: a central conserved domain flanked and two hypervariable domains (upstream and downstream). Three conserved sequence blocks (CSBs) were detected at the central conserved domain (CSB-F, CSB-E, CSB-D) as well as at the downstream hypervariable region (CSB1, CSB2, CSB3) (Fig. 3). Although additional CBSs have been identified for the central conserved domain (CSB-B, CSB-C) in mammals [46], the three identified herein for the species *A. altior* and *A. bacalarensis* are those commonly found in fishes [47–49]. The upstream hypervariable domain in the CR of mitogenome of *A. altior* and *A. bacalarensis* has a length of 342 bp and includes seven copies of the motif TACAT and seven copies of the palindromic motif ATGTA. A change in the motif sequence (TGCAT) was observed in *S. chuatsi* but not in either mitogenome of *Astyanax*. Compared to those from the central conserved domain, CSBs in the downstream hypervariable domain displayed larger variation across the three fish species compared. Notably, CSB2 and CSB3 were slightly more variable than CSB1.

### Phenetic patterns of genetic variation

**Fig. 4:**
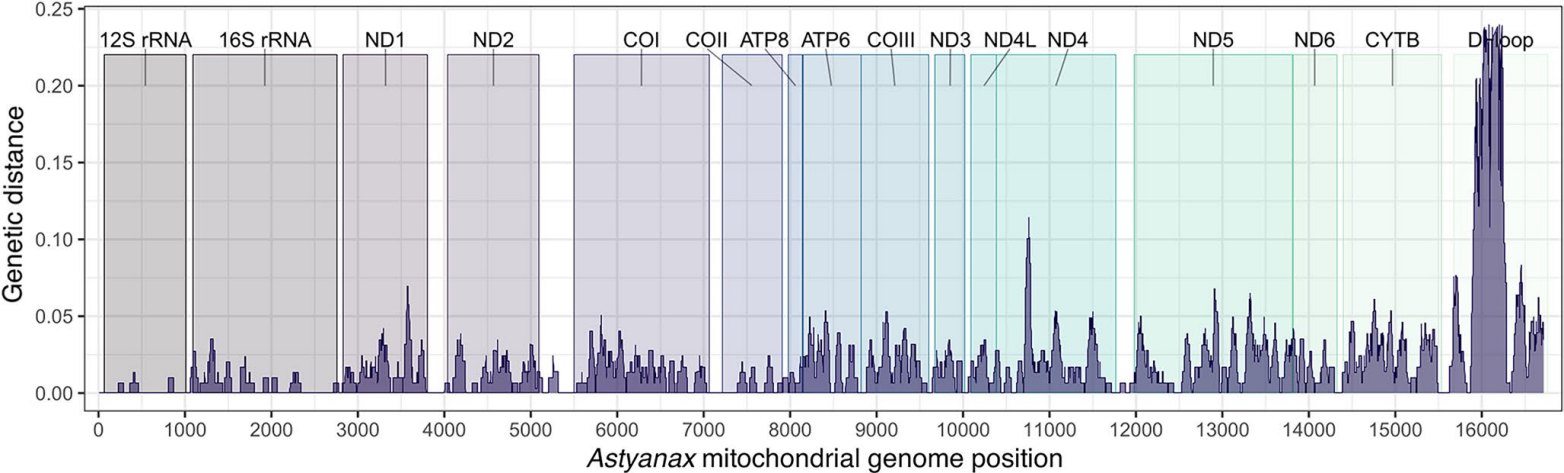
Kimura-2-parameter (K2P) distances for 60 bp windows from the complete mitochondrial genomes of the Middle American species of *Astyanax s.s.* sampled in this study (*A. aeneus*, *A. mexicanus*, *A. altior*, and *A. bacalarensis*). Colored boxes highlight rRNAs, coding genes, and the control region. Variations of peak heights indicate the distance related to the base mitogenome of *A. altior*

Mitogenome-wide pairwise genetic distances among sampled Middle American species of *Astyanax* ranged from 0.3 to 2.2%, regardless of correction (i.e., K2P and *p*-distances were essentially the same) (Table 2), being smallest between *A. altior* and *A. bacalarensis* and largest between either and *A. mexicanus*. The degree of genetic differentiation between the mitogenomes of *A. altior* and *A. bacalarensis* (0.3%) is lower than that between conspecific samples of *A. mexicanus* (1.1%). A similar pattern of genetic variation was observed when considering PCGs only (Table 2). As expected, our graphical analysis of variation by gene/region (Fig. 4) revealed that CR exhibits the highest number of polymorphisms and overall variation (least conserved), followed by *ND4L*, *ND4*, *ND5* and *CYTB*. Except for *ATP8*, most of the remaining PCGs exhibit intermediate levels of variation. Conversely, variation is lowest in the rRNA ribosomal genes.

**Table 2:** Mitogenome-wide pairwise genetic distances (in percentages) among sampled middle American species of *Astyanax*. K2P and *p*-distances were essentially the same. Based on PCGs in parenthesis if different than for the entire mitogenome. Codes after species names correspond to mitogenome GenBank accession numbers

	*A. altior* PV765696	*A. bacalarensis* PV765695	*A. aeneus* BK013055	*A. mexicanus* AP011982	*A. mexicanus* BK013062
***A. altior*** PV765696	**0**	0.3	0.018 (0.015)	2 (2.1)	2.2 (1.9)
***A. bacalarensis*** PV765695	0.3	**0**	0.017 (0.015)	2 (2.1)	2.1 (1.9)
***A. aeneus*** BK013055	1.7 (1.5)	1.7 (1.4)	**0**	2.1	2.2 (1.8)
***A. mexicanus*** AP011982	2	2 (2.1)	0.02	**0**	1.1 (1.3)
***A. mexicanus*** BK013062	2.2 (1.8)	2.2 (1.9)	2.2 (1.8)	1.1 (1.3)	**0**

### Phylogenetic relationships and species limits

**Fig. 5:**
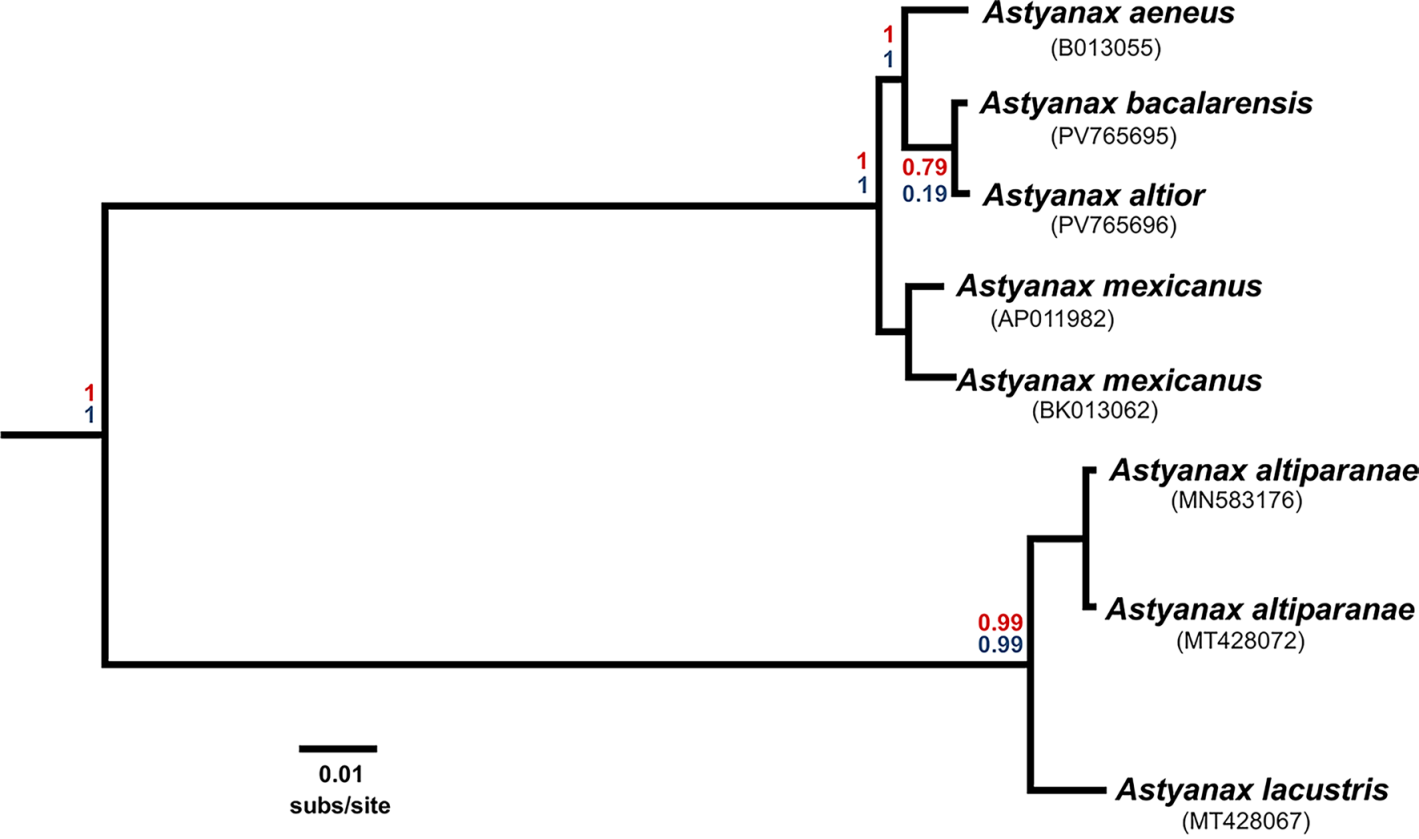
Phylogenetic relationships among species of *Astyanax s.s.* sampled in this study (ingroup). Results from the Bayesian inference of phylogeny under the multispecies coalescent model as implemented in bpp. Phylogeny inferred from comparative mitochondrial PCGs from relevant available mitogenomes and the newly generated herein for *A. altior* and *A. bacalarensis*. The topology was the same regardless of Bayesian inference method (MrBayes or bpp). Outgroup taxa not shown. Numbers in parentheses under each taxon name correspond to the respective mitogenome’s GenBank accession number. Values at nodes correspond to posterior probabilities from the bpp species delimitation analyses (A10 in red; A11 in blue). All nodes in the tree inferred with MrBayes resulted in clade credibility values of 1

Regardless of inferential method, the resulting phylogeny (Fig. 5) is completely resolved and strongly supported, with the five valid species of *Astyanax s.s.* sampled for phylogenetic analyses (*A. altior*, *A. bacalarensis*, *A. aeneus*, *A. mexicanus*, and *A. lacustris*) forming a robust monophyletic group in which *A. lacustris* (*cis*-Andean, South American) (inclusive of *A. altiparanae*) is sister to the remaining species (*trans*-Andean, Middle American). Within the Middle American clade, *A. mexicanus* is resolved as sister to a clade consisting of *A. aeneus* and the sister pair *A. altior* + *A. bacalarensis*. Like with the phenetic analyses, the phylogenetic differentiation between *A. altior* and *A. bacalarensis* is minimal, as evidenced by the very short branch lengths connecting the two.

Species delimitation with bpp resulted in very similar outputs regardless of analysis type (A10 and A11), indicating that the data strongly supports the inferred topology and that the input tree (MrBayes-inferred or bpp-co-estimated) did not bias the results of the species delimitation analysis. Notably, and in disagreement with the current taxonomy, both bpp analyses rejected the hypothesis that *A. bacalarensis* and *A. altior* are different species, as evidenced by the considerably low PPs (A10 = 0.79; A11 = 0.19) for the node containing the sister pair *A. altior* + *A. bacalarensis*. Similarly, these results support the distinction of *A. altiparanae* from *A. lacustris*, implying that these lineages correspond to different species. In contrast to these findings, the resulting species delimitation scheme offered strong support for the recognition of *A. aeneus* and *A. mexicanus* as distinct and valid species.

## Discussion

The importance of complete mitochondrial genomes in evolutionary biology research cannot be overstated, for they have become a powerful source of genetic information in comparative studies in the fields of taxonomy (i.e., species identification and delimitation), phylogenetics (i.e., tree reconstruction and molecular dating), population genetics and phylogeography (i.e., analysis of intraspecific genetic variation and its distribution in geographic space), and genome evolution, among others [50–53]. Among the reasons why the mitogenome is suitable for these types of studies are its maternal inheritance and lack of recombination (making it easier to trace lineages while avoiding potentially confounding issues of paralogy), high mutation rate (improving resolution at recent divergences), conserved structure (allowing comparisons between distantly related taxa), and small size (making it easier and cheaper to sequence, assemble, and annotate) [53, 54]. Fish systematics has unarguably benefited from the generation and analysis of comparative mitogenomic data, resulting in phylogenetic hypotheses at various levels of divergence aimed at addressing taxonomic and biogeographic questions in many groups of fishes [15, 30, 55–58].

While it has been argued that, for many taxonomic groups (such as fishes), publication of new mitogenomes is no longer contributing significantly to the field, and that a lot of mitochondrial genome papers are purely descriptive and do not address a specific hypothesis or research question [59], we reckon this is neither the case for *Astyanax* nor for the present study. Despite their diversity, species of the genus *Astyanax* are barely represented in terms of published complete mitochondrial genomes. Such a lack of comparative mitogenomic data hinders progress in our understanding of fundamental questions that could be addressed using this type of data. Sequencing, assembly, and annotation of the novel mitogenomes of *Astyanax* presented herein therefore adds to an expanding catalogue of genomic comparative data and genetic resources with potential to be used in manifold evolutionary studies on this group of fishes and even extending to more inclusive clades. Furthermore, in addition to the descriptive component inherent to our study, we used these novel mitogenomic data in a comparative framework to address, at least preliminarily, an explicit evolutionary question regarding the systematics of *Astyanax* (i.e., Are *A. altior* and *A. bacalarensis* really different species?).

Characins of the genus *Astyanax* are one of the most taxonomically challenging groups of Neotropical freshwater fishes, and although a recent systematic study contributed to unraveling the historical taxonomic mess by redefining its limits and composition [1], uncertainties remain regarding the taxonomic status and relationships of some included species, particularly within the Middle American clade. In relation to these uncertainties, Terán et al. [1] raised issues with some of the species’ descriptions and diagnoses offered by Schmitter-Soto [10] in his revision of Central and North American species, particularly to Schmitter-Soto’s [10] overreliance on osteological characters that tend to be highly variable in other, closely related characins. While the validity of *A. altior* and *A. bacalarensis* had not been explicitly questioned in the published literature, scrutiny of the diagnostic characters proposed by Schmitter-Soto [10] to distinguish between them, reveals that these are, for the most part, either overlapping or weak (i.e., rather variable). Furthermore, differences in body depth, the leading diagnostic character purportedly telling these two species apart, might be related to plastic variation instead of character fixation after speciation, as suggested in a recent unpublished undergraduate thesis [60]. Using comparative morphometric data from numerous populations of *A. altior* and *A. bacalarensis* across the YP, this thesis concluded that, despite some significant differences in overall body shape between putative species, the observed variation is not substantial and may simply reflect phenotypic plasticity in response to environmental variation, rendering the taxonomic distinction proposed by Schmitter-Soto [10] suspect at best.

The present study therefore constitutes the first attempt at testing the distinction between *A. altior* and *A. bacalarensis* (and in turn, their validity) based on comparative molecular data. Distance-based methods were initially used to assess the degree of genetic divergence between these putative species. With the bpp analyses we aimed to test whether the current taxonomy corresponds to the species limits entailed by our mitogenomic dataset under a coalescent approach and ultimately shed light on the species status of *A. altior* and *A. bacalarensis.* Overall, our results (phenetic and phylogenetic) strongly suggest that, as currently defined and delimited, these species might correspond to the same metapopulation lineage and would therefore represent synonyms. Mitogenome-wide pairwise genetic distances between the sampled individuals of *A. altior* and *A. bacalarensis* were negligible (0.3%), indicating almost identical mitogenomes. Notably, this value is even smaller than the observed genetic distance between the sampled conspecifics of *A. mexicanus* (1.1%), and in contrast to the observed interspecific divergences among the sampled Middle American species of *Astyanax* (~ 2%) (Table 2). Echoing this pattern, the degree of sequence divergence in the Cytochrome Oxidase I (COI) mitochondrial gene—the DNA barcode in animals—between *A. altior* and *A. bacalarensis* was only ~ 0.4% (six variable sites in total), a value much lower than the traditionally employed ~ 3% heuristic threshold for conspecifics in most teleost fishes and other vertebrates [61–65]. The uncovered minimal divergence in mtDNA between *A. altior* and *A. bacalarensis* therefore does not appear to be consistent with their species-level distinction. Since our bpp analyses rejected the hypothesis that *A. altior* and *A. bacalarensis* are different species (Fig. 5), our phylogenetic results corroborate the findings derived from distance-based methods.

While not the main objective of our study, our results also shed light on the relationships among Middle American species of *Astyanax* and the phylogenetic position of *A. altior* and *A. bacalarensis* with respect to their regional congeners. The resulting phylogeny places *A. aeneus* as sister to the clade *A. altior* + *A. bacalarensis*, an unsurprising result given that populations of *Astyanax* from the YP were at some point considered part of a more encompassing *A. aeneus* [10, 66, 67]. Contrary to the findings of a more comprehensive study focused on the evolutionary history of Middle American *Astyanax* [66], our bpp species delimitation results support the distinction of *A. aeneus* as a separate species-level lineage, different from the species found in the YP (Fig. 5). The phylogenetic placement of *A. mexicanus* implied by our results, conversely, is compatible with the findings of that previous study [66], which places *A. mexicanus* as sister to an expanded *A. aeneus* including populations from cenotes of the YP. Our recognition of *A. aeneus* as a separate species-level lineage from the clade *A. altior* + *A. bacalarensis*, however, should be interpreted with caution given our rather limited geographic sampling (i.e., only one individual representing the geographic diversity of most sampled species). Hopefully future research could address this limitation.

Although only tangential to our main research question, our taxonomic sampling and species delimitation analyses allowed testing the hypothesis that the South American species *A. lacustris* and *A. altiparanae* are synonyms (the former its valid name and the latter its junior synonym), as proposed in a revisionary study of the *Astyanax bimaculatus* “caudal peduncle spot” subgroup from La Plata and São Francisco Rivers drainages [42]. Notably, the results of our species delimitation analysis (Fig. 5) support the distinction of *A. altiparanae* and *A. lacustris* as different species, thus refuting the abovementioned synonymy and independently validating a recent proposal to resurrect *A. altiparanae* on the basis of mtDNA, cytogenetic, and morphometric data [68]. Although we considered important to address how our results fit within the framework of this taxonomic controversy, commenting further on this matter is beyond the scope of this study.

## Conclusions

In addition to generating and reporting novel mitogenomic data for a highly diverse and taxonomically challenging group of Neotropical fishes with minimal representation in genomic repositories and databases, we were able to use these novel data in a comparative phylogenetic framework to test, for the first time, the evolutionary (and therefore taxonomic) distinctiveness of the species of *Astyanax* known to occur in cenotes of the YP in southern Mexico. Although our results, based on sound analyses of comparative mitogenome-wide data, strongly suggest that the species *A. altior* and *A. bacalarensis* correspond to the same species-level lineage, we defer synonymizing them until a proper revisionary study—based on analysis of both morphological/anatomical and genetic data from a larger sample of taxonomic and geographic diversity—corroborates our findings. Such a study should ideally include a larger sampling of populations from across the YP as well as from closely related and biogeographically relevant species, such as *A. aeneus* (Southwestern North America and Middle America), *A. caballeroi* (Catemaco Lake basin), *A. baileyi* (Usumacinta River basin), *A. petenensis* (Lake Petén basin, Guatemala, and adjacent Chiapas, southern Mexico), *A. brevimanus* (Southern North America and Middle America), *A. rioverde* (Mexico, Atlantic versant), and *A. mexicanus* (Southern North America and Pacific versant of Mexico).

## Electronic supplementary material

Below is the link to the electronic supplementary material.

Supplementary Material 1

Supplementary Material 2

## Data Availability

Novel comparative sequence data (complete mitochondrial genomes) that support the findings of this study have been deposited in GenBank: *Astyanax altior* (GenBank accession number PV765696) and *Astyanax bacalarensis* (GenBank accession number PV765695). Raw data are available as an NCBI (https://www.ncbi.nlm.nih.gov/bioproject/) BioProject (PRJNA1276058). FastQC quality reports in HTML format (clean data), as well as Geneious assemblies’ reports, are available in the free, open-source, general-purpose data repository Zenodo (https://www.zenodo.org) (10.5281/zenodo.15647728).
