# Supplementary figures and images for: The complete mitochondrial genomes of the two species of Astyanax (Characiformes: Acestrorhamphidae) that occur in cenotes of the Yucatán Peninsula karst aquifer: comparative analyses and their taxonomic implications
Source: Mol Biol Rep. 2025 Jul 10;52(1):698. doi: 10.1007/s11033-025-10788-6 (PMC12246002; doi:10.1007/s11033-025-10788-6)

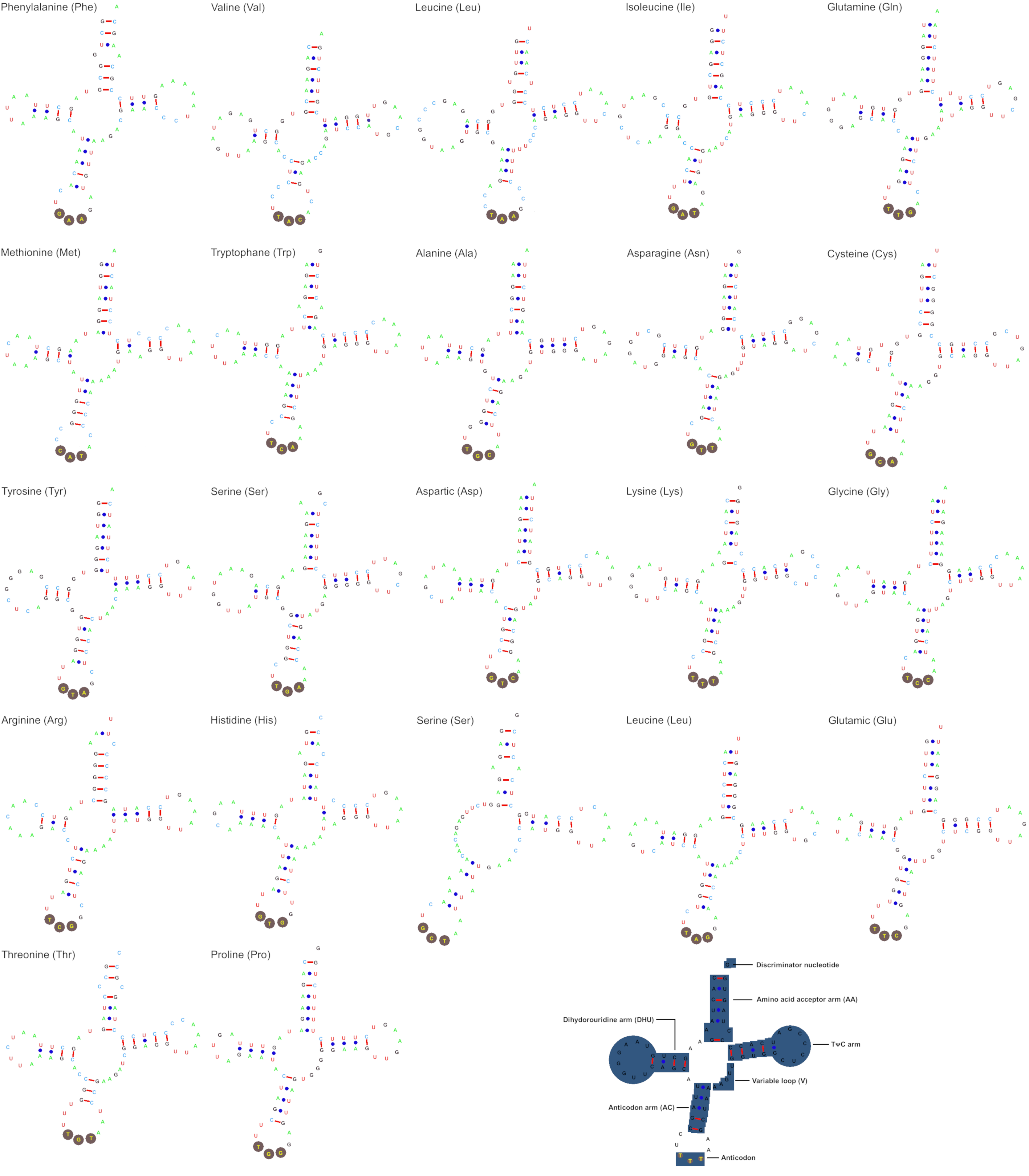

Supplement: Supplementary file 1 — Supplementary Material 1 [file 11033_2025_10788_MOESM1_ESM.tif]

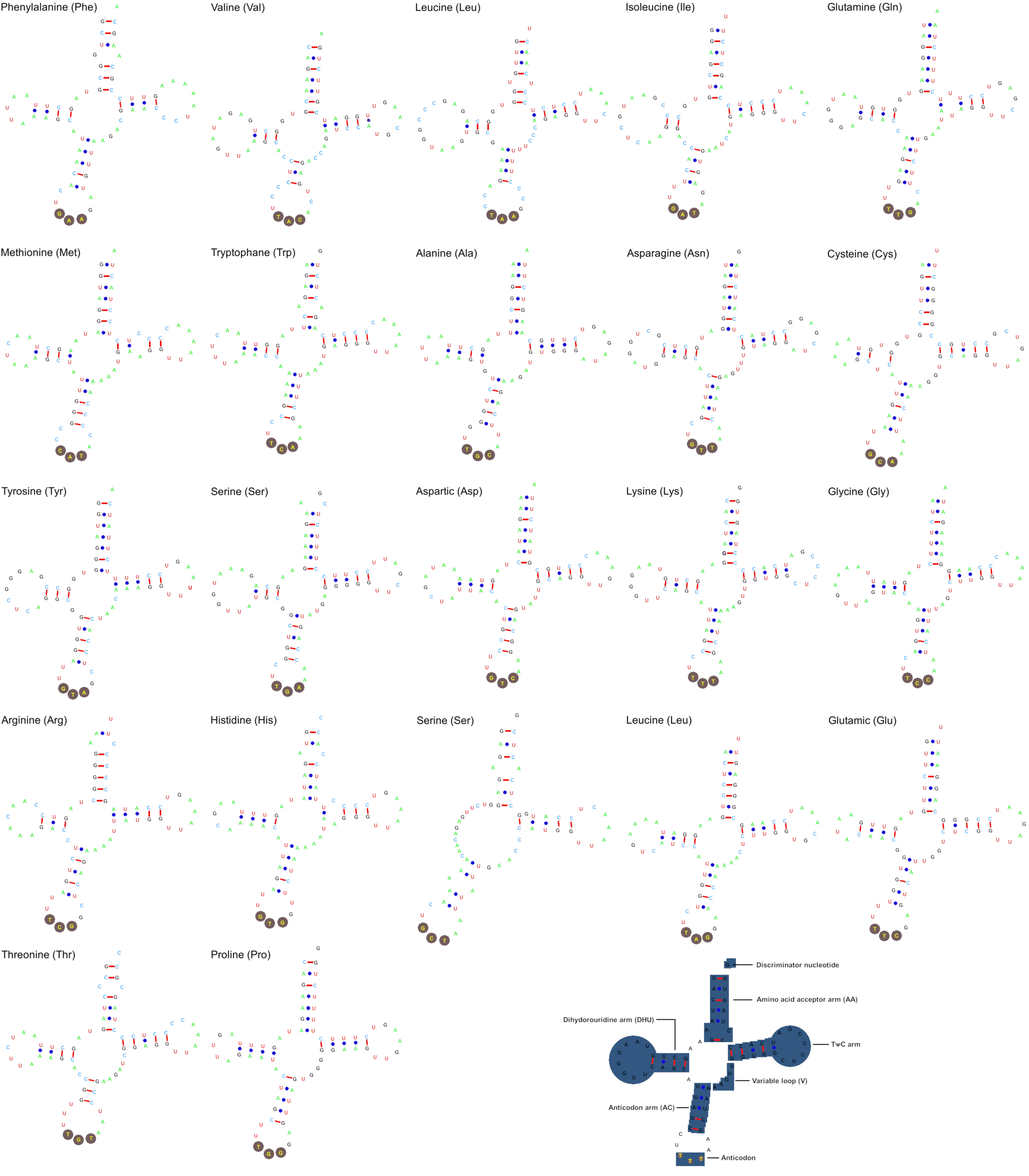

Supplement: Supplementary file 2 — Supplementary Material 2 [file 11033_2025_10788_MOESM2_ESM.tif]
